# Genetic diversity and genome-scale population structure of wild Indian major carp, *Labeo catla* (Hamilton, 1822), revealed by genotyping-by-sequencing

**DOI:** 10.3389/fgene.2023.1166385

**Published:** 2023-05-09

**Authors:** Bismay Sahoo, Gargee Das, Priyanka Nandanpawar, Nirjharini Priyadarshini, Lakshman Sahoo, Prem Kumar Meher, Uday Kumar Udit, Jitendra Kumar Sundaray, Paramananda Das

**Affiliations:** Fish Genetics and Biotechnology Division, ICAR-Central Institute of Freshwater Aquaculture, Bhubaneswar, India

**Keywords:** single nucleotide polymorphisms (SNPs), genotyping-by-sequencing (GBS), *Labeo catla*, population genomics, genetic differentiation

## Abstract

*Labeo catla* (catla) is the second most commercially important and widely cultured Indian major carp (IMC). It is indigenous to the Indo-Gangetic riverine system of India and the rivers of Bangladesh, Nepal, Myanmar, and Pakistan. Despite the availability of substantial genomic resources in this important species, detailed information on the genome-scale population structure using SNP markers is yet to be reported. In the present study, the identification of genome-wide single nucleotide polymorphisms (SNPs) and population genomics of catla was undertaken by re-sequencing six catla populations of riverine origin from distinct geographical regions. DNA isolated from 100 samples was used to perform genotyping-by-sequencing (GBS). A published catla genome with 95% genome coverage was used as the reference for mapping reads using BWA software. From a total of 472 million paired-end (150 × 2 bp) raw reads generated in this study, we identified 10,485 high-quality polymorphic SNPs using the STACKS pipeline. Expected heterozygosity (He) across the populations ranged from 0.162 to 0.20, whereas observed heterozygosity (Ho) ranged between 0.053 and 0.06. The nucleotide diversity (π) was the lowest (0.168) in the Ganga population. The within-population variation was found to be higher (95.32%) than the among-population (4.68%) variation. However, genetic differentiation was observed to be low to moderate, with F_st_ values ranging from 0.020 to 0.084, and the highest between Brahmani and Krishna populations. Bayesian and multivariate techniques were used to further evaluate the population structure and supposed ancestry in the studied populations using the structure and discriminant analysis of principal components (DAPC), respectively. Both analyses revealed the existence of two separate genomic clusters. The maximum number of private alleles was observed in the Ganga population. The findings of this study will contribute to a deeper understanding of the population structure and genetic diversity of wild populations of catla for future research in fish population genomics.

## 1 Introduction

*Labeo catla* (Hamilton, 1822), popularly known as “catla,” is the second most popular Indian major carp (IMC) (Murthy, 2002). This potamodromous fish is indigenous to the Indo-Gangetic riverine system of India and the rivers of Bangladesh, Nepal, Myanmar, and Pakistan (Reddy, 2005). It is widely cultured in polyculture systems of the Indian subcontinent because of its fast-growing nature, compatibility with other major carps, unique surface feeding behavior, good taste, and consumer preference (FAO, 2006–2020). At present, *L. catla* constitutes ∼3.4% of the global freshwater aquaculture production ($5 billion USD) (FAO, 2019). In the last 20 years, the culture of IMCs has intensified due to their high commercial value, increasing demand, and fast growth rate with 4,713,340 tons of production (FAO, 2000-2020). An extreme population decline trend of 87% in the wild capture of *L. catla* species has been observed by comparing the survey data from 1958 to 1994 (Payne et al., 2004) in Indian rivers, which may have been caused by overfishing and habitat alterations by construction of dams and pollution (Reddy, 2005). Wild populations are used as a source of genetic diversity for hatchery stock. To establish effective management and conservation guidelines, as well as develop genetic improvement programs for catla species, it is crucial to first assess genetic variations among populations using extensive information on the genetic structure of the wild populations of catla.

However, quite limited genetic information on studies pertaining to genetic variation in wild populations of catla is available using microsatellites (Alam and Islam, 2005; Ahmed and Abbas, 2018; Faroque et al., 2021), random amplified polymorphic DNA (RAPD) (Islam et al., 2005; Rahman et al., 2009), and mitochondrial DNA markers (Das et al., 2012; Sarmah and Sarmah, 2016; Behera et al., 2017). In addition to this, very few studies on genetic diversity employing SNP markers have been reported in the wild populations of catla (Hamilton et al., 2019). Nevertheless, the degree, pattern, and variation of the genetic structure in the present context are still poorly understood.

Being the most abundant molecular marker in the genomes of various vertebrate organisms, SNPs have been the markers of choice for a variety of applications, including population genomics. Over the last two decades, next-generation sequencing (NGS) technology has evolved, enabling the rapid and inexpensive identification of genome-wide SNP markers in any organism without access to prior genomic data. Furthermore, genotyping-by-sequencing (GBS) has become a potential genome-wide genotyping tool that employs an enzyme-based complexity reduction method to simultaneously discover and genotype molecular markers across the entire genome, with or without a reference (Elshire et al., 2011). The genome-wide SNPs discovered using the GBS technique have formed the basis of population structure and diversity studies (Nyinondi et al., 2020; Yang et al., 2020; Fagbemi et al., 2021), linkage map construction (Everett and Seeb, 2014; Uchino et al., 2018; Yin et al., 2020), QTL analyses (Uchino et al., 2018), genome-wide association studies for economically important traits, such as disease resistance (Correa et al., 2015; Gutierrez et al., 2015; Wang et al., 2017), and the application of genomic selection (Wolc et al., 2015; Wang et al., 2019; Luo et al., 2021; Luo et al., 2022; Wang et al., 2022).

In the present investigation, genome-wide SNPs were identified and population genomics analysis was performed in *L. catla* by genotyping-by-sequencing of six riverine catla populations from distinct geographical regions of India. The results of this study will enhance our knowledge in understanding genetic diversity and population structure and help in the conservation and management of wild populations of catla.

## 2 Materials and methods

### 2.1 Sample collection

**FIGURE 1:**
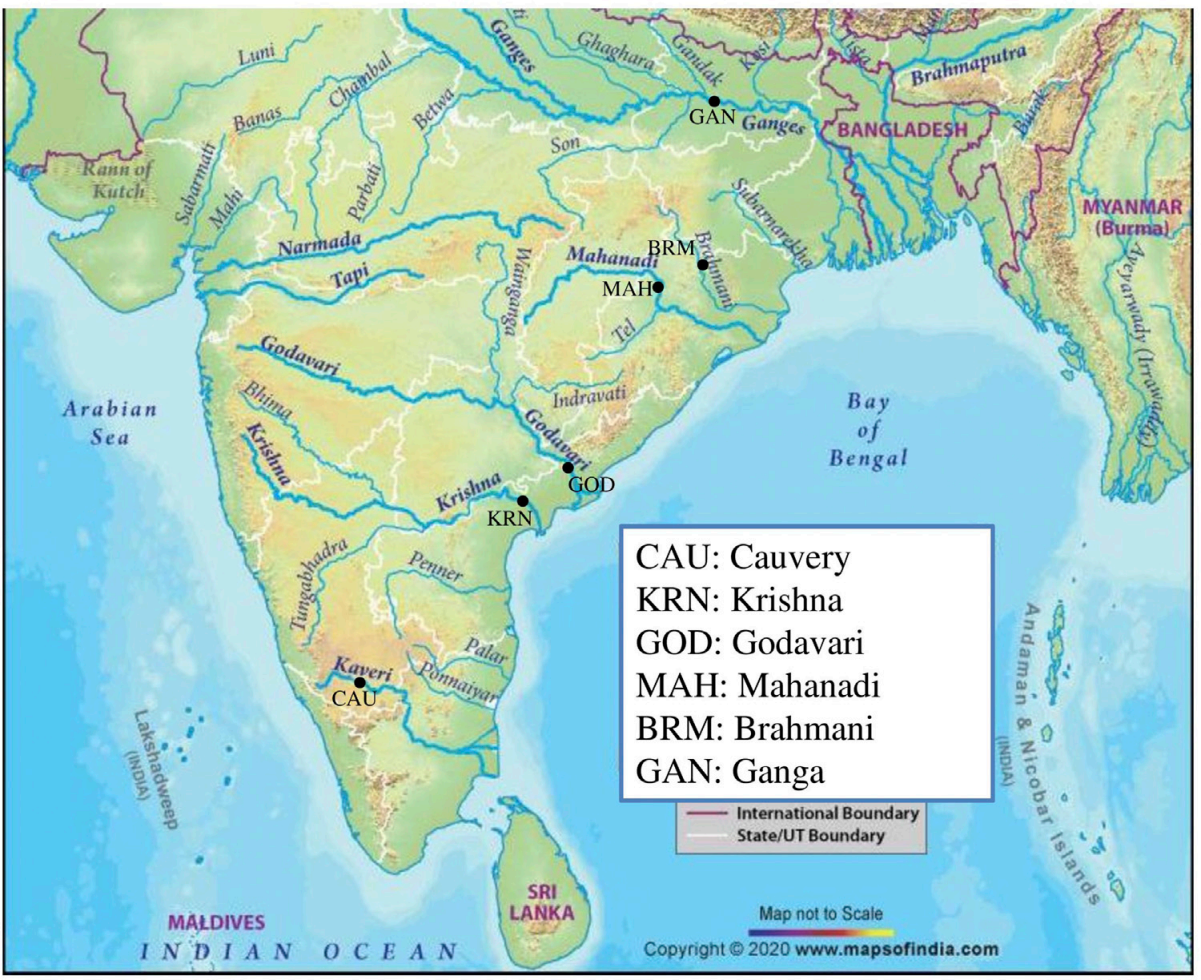
Sampling locations of six catla populations, sorted by latitude from the South.

Fish samples pertaining to riverine populations were collected from six Indian rivers viz, Cauvery (CAU), Krishna (KRN), Godavari (GOD), Mahanadi (MAH), Brahmani (BRM), and Ganga (GAN) (Table 1; Figure 1). The waters of the Cauvery River at Mysore are reported to have high metal contamination and altered water quality due to nearby agricultural and industrial activities (Susheela et al., 2014), as well as a damming effect due to the presence of the Krishna Raja Sagara Dam. Fish sampling from the Krishna River was performed at Vijayawada, where compromised water quality due to fly ash arising from the Vijayawada Thermal Power Station is reported (Rao and Ravindhranath, 2013). Moreover, the fish fauna of the Krishna River near the Prakasam Barrage is threatened due to several factors, including heavy harvesting of fish resources, competition and predation by introduced species, and habitat degradation due to pollutants (Jadhav et al., 2011). Rajahmundry was the only sampling site where the *Water Quality Index (WQI)* of the Godavari River was found to be fair (Sravya et al., 2017) and less environmental impact was observed. The Mahanadi River near Sambalpur in Odisha state is a major site for coal fields, while the Brahmani River near Barkote is moderately affected by industrial and agricultural runoffs (Nath et al., 2018). Samples were collected from the Ganga River at Patna, a state capital witnessing a colossal level of urbanization (Imam. 2020) along with air and water pollution due to rampant sand mining and brick kilns. Recently, a report on the shifting of the Ganga River by an average of 0.14 km per year from the city of Patna has alerted environmentalists to focus on its associated flora and fauna (Kumar et al., 2014).

**TABLE 1:** Origin of catla (*Labeo catla*) populations.

Population/rivers	Sampling site	No. of individuals	Latitude	Longitude	Sampling year
Cauvery (CAU)	Mysore	20	12.528591	75.993629	2011
Krishna (KRN)	Vijayawada	7	15.754239	80.897270	2011
Godavari (GOD)	Rajahmundry	26	16.708548	82.118683	2010
Mahanadi (MAH)	Sambalpur	24	21.012094	84.040789	2009
Brahmani (BRM)	Barkote	8	21.371887	85.777592	2010
Ganga (GAN)	Patna	15	25.59410	85.13760	2020

Samples were collected from adult individuals ranging from 1 to 2 kg in weight. Natural hybrids of catla are known to be rare in the riverine populations. Even then, all sampled individuals were checked phenotypically, considering the shape of the head and mouth (Sarder et al., 2011). As the individuals ranged from 1 to 2 kg in weight, they were easily distinguishable. No hybrids were detected. Fin clips from 100 individuals were stored at −20°C in 95% ethyl alcohol until DNA extraction. All handling of fish was carried out following the guidelines for control and supervision of experiments on animals established by the Government of India and approved by the Institutional Animal Ethics Committee (AEC) of ICAR-CIFA.

### 2.2 DNA isolation

High-molecular weight (MW) genomic DNA (gDNA) was extracted from the fin clips using a “DNeasy Blood and Tissue Kit” (QIAGEN, Hilden, Germany), according to the manufacturer’s protocol. The integrity and concentration of the DNA were checked by 0.8% agarose gel electrophoresis and using a Nanodrop Biospectrometer (Eppendorf India Pvt. Ltd.). Samples with an adequate concentration (≥100 ng/μl) and quality (intact high-MW DNA) were selected. One GBS library was prepared using 100 samples.

### 2.3 GBS library construction and sequencing

Using Elshire et al.’s (2011) methodology with modifications, GBS data were generated. In brief, each 200 ng of DNA sample was digested with five units of the ApeK1 enzyme at 75°C for 4 h and cooled down to 4°C, followed by GBS library preparation. Unique barcodes and a “common” adapter were used for each individual ligation. After ligation, they were pooled and purified using AMpure DNA purification beads and the genomic fragments were then amplified in 50-µl volumes. GBS library purification was carried out using Agencourt AMPure XP SPRI magnetic beads (Beckman Coulter). The quality of the GBS library was assessed using the 4150 TapeStation System (Catalog: G2992AA, Agilent). The GBS library was sequenced using the Illumina HiSeq 2000 platform with paired-end (PE) reads of 150 × 2 bp.

### 2.4 Sequence data analysis and SNP genotyping

The quality of the data was checked with FastQC v0.11.8 (Andrews, 2010) and MultiQC v1.9 (Ewels et al., 2016) software. The fastp (Chen et al., 2018) program was used to remove the adapter, along with duplicate sequences and bases of poor quality (Q < 30) from the raw reads. The high-quality reads of each individual were mapped to the catla reference genome [GeneBank acc. no: GCA_012976165.1 (Sahoo et al., 2020)] using BWA v0.6.12 software with the “MEM” option (Li, 2013) and default parameters. The reference catla genome had an assembled genome size of 1.01 Gb, 5,245 scaffolds and >92% BUSCO completeness. SAMtools v0.1.19 (Li et al., 2009) was used to convert the SAM files into BAM and sort alignments by genomic coordinates. SNPs were discovered using the GSTACKS program with a default model (Marukilow) in STACKS v2.59 (Catchen et al., 2013; Rochette et al., 2019) with the parameters var-alpha = 0.05, gt-alpha = 0.05 (both default), and min-mapq = 30, to reduce the impact of misplaced reads. Subsequently, SNPs present in at least 75% of the individuals in each population (r) and across populations (R) were identified with a minor allele frequency of 0.05 using the POPULATIONS program of STACKS. Additionally, these SNPs were filtered again with mean depth >= 5 using VCFtools v0.1.12 (Danecek et al., 2011) (Table 2). The resultant SNPs were used for subsequent genetic analysis.

**TABLE 2:** Summary of identified SNPs across six populations.

SNP details	No. of filtered SNPs
SNP discovery (min-mapq = 30; var-alpha = 0.05; gt-alpha = 0.05)	655,548
SNP filtered (maf = 0.05; *p* = 6; R = 0.75; r = 0.75)	26,348
Final SNP (mean depth >=5)	10,485

### 2.5 Genetic diversity analysis and effective population size

STACKS v2.59 (Rochette et al., 2019) software was used to assess the genetic variation metrics, such as mean expected heterozygosity (H_e_) and observed heterozygosity (H_o_), average inbreeding coefficients (F_IS_), nucleotide diversity (π) with their standard errors (SE), mean frequency of the most frequent allele (P), Hardy–Weinberg equilibrium (HWE) estimates, and pairwise F_st_ values across the six populations of catla. The pairwise F_st_ heatmap was constructed with Heatmapper software (Babicki et al., 2016). Furthermore, analyses of molecular variance (AMOVA) were performed to find out the distribution of genetic variations using Arlequin v3.5 software (Excoffier and Lischer, 2010). The effective population size (Ne) was estimated by using a molecular co-ancestry method (Nomura, 2008), as implemented in NeEstimator v2.01 (Do et al., 2014). PGDSpider software (Lischer and Excoffier, 2012) was used to convert the vcf file into a GENEPOP input file for NeEstimator.

### 2.6 Clustering and ancestry analysis

For visualization and insight into the existence of genetic clusters, principal component analysis (PCA) was carried out using the R tool v3.5 (R core team, 2018) with the Adegenet v2.1.1 package (Jombart, 2008; Jombart and Ahmed, 2011). The scatter plot was created in R using the “ggplot2″ package (Ginestet et al., 2011) with the first and second components. Thereafter, the genetic structure was analyzed through the multivariate approach called DAPC (Jombart et al., 2010) in the same R package that utilizes the number of principal components and discriminant axes to divide the sample into between-group and within-group components in order to maximize discrimination across populations. In DAPC, data are first converted using principle component analysis (PCA), and then, clusters are determined using discriminant analysis (DA). This approach offers a visual representation of the genetic difference in multivariate space among populations. Additionally, the probable population admixture was analyzed using Structure v.2.3.4 software (Hubisz et al., 2009) under correlated allele frequencies and admixture models (settings: K values = 1–10; burn-in length = 50,000; MCMC replicates = 200,000; iterations = 10). L(K) and ΔK statistics were inferred in both STRUCTURE HARVESTER (Earl and vonHoldt, 2012) and CLUMPAK (Kopelman et al., 2015) software applications to select the optimal number of genetic clusters (K). A UPGMA tree was also constructed to cluster the six catla populations using the R tool in the “ape” v5.6.2 (Paradis and Schliep, 2019) package.

### 2.7 Linkage disequilibrium

TASSEL v5.0 (Bradbury et al., 2007) software was used to analyze LD (r^2^) for the six catla populations with a sliding window size of 50. A non-linear regression curve was used to estimate LD decay, and the distance between SNPs at which the average r^2^ drops to 50% of the maximal LD value was used to compute the LD decay distance.

## 3 Results

### 3.1 SNP identification and genotyping

GBS libraries were sequenced, generating a total of 472 million raw data with 0.71 Gb of bases on average for each individual. Each randomly selected read was mapped to the sequences of various fish species including *Cyprinus carpio* and *L. catla* in the NCBI NT database, demonstrating the reliability of the reads. After removing reads with low base quality (Q < 30), on average, 96.91% of filtered reads were successfully mapped to a catla reference genome with approximately 278 million clean data (Supplementary Table S1), and an average of 2.78 million clean reads per individual were retained. After applying the filtering criteria that a locus must be present in more than 75% of the individuals in a population and across populations, the MAF must be greater than 0.05, and the mean depth must be >= 5, 10,485 putative SNP markers were selected for further genetic data analysis. The average depth of the 10,485 SNPs was 10. The STACKS output indicated the presence of 8,963 loci and 1–2 SNPs per locus.

### 3.2 Genetic diversity and population structure

In this study, the genetic diversity of six catla populations and the allele polymorphism of these groups were analyzed. Across all populations, the expected heterozygosity (H_e_) ranged from 0.162 (GAN) to 0.20 (GOD) and observed heterozygosity (H_o_) varied between 0.053 (GAN) and 0.06 (BRM). The nucleotide diversity (**π**) varied from 0.168 to 0.205 across the six populations, with the GAN population having the least value. The mean frequency of the most frequent allele (P) varied from 0.87 to 0.89 among the six populations of catla. The inbreeding coefficients (F_IS_) of the six populations of catla ranged from 0.382 to 0.637 and were highest in the GOD population (Table 3). Six populations with 77,216 to 78,421 loci out of 79,861 loci showed substantial deviations from the Hardy–Weinberg equilibrium (*p* < 0.05). Maximum numbers of private alleles were observed in the GAN population in comparison to other studied wild populations. Ne values were successfully estimated for 10,485 SNP markers with the highest value of 1.2 in the KRN population, whereas the lowest value was 0.8 in the CAU population (Supplementary Table S2).

**TABLE 3:** Genetic variability and allele heterogeneity in six populations of *Labeo catla*.

Population	H_e_ (SE)	H_o_ (SE)	F_IS_ (SE)	π (SE)	HWE	P	Private alleles
CAU	0.173 ± 0.0012	0.055 ± 0.0016	0.570 ± 0.0101	0.178 ± 0.0013	77,768	0.887	2
KRN	0.175 ± 0.0016	0.059 ± 0.0017	0.382 ± 0.0043	0.190 ± 0.0018	76,851	0.876	0
GOD	0.200 ± 0.0012	0.059 ± 0.0016	0.637 ± 0.0153	0.205 ± 0.0012	77,677	0.870	17
MAH	0.170 ± 0.0013	0.054 ± 0.0016	0.543 ± 0.0152	0.174 ± 0.0014	77,911	0.888	19
BRM	0.179 ± 0.0017	0.060 ± 0.0017	0.384 ± 0.0069	0.194 ± 0.0018	77,216	0.874	0
GAN	0.162 ± 0.0015	0.053 ± 0.0016	0.428 ± 0.0099	0.168 ± 0.0016	78,421	0.890	22

Note: He denotes expected heterozygosity, Ho denotes observed heterozygosity, π denotes nucleotide diversity, and F_IS_ denotes the inbreeding coefficient with SE. HWE denotes the number of loci significantly deviating from the Hardy–Weinberg equilibrium (*p* < 0.05). P denotes the population’s mean frequency of the most common allele at each locus.

**FIGURE 2:**
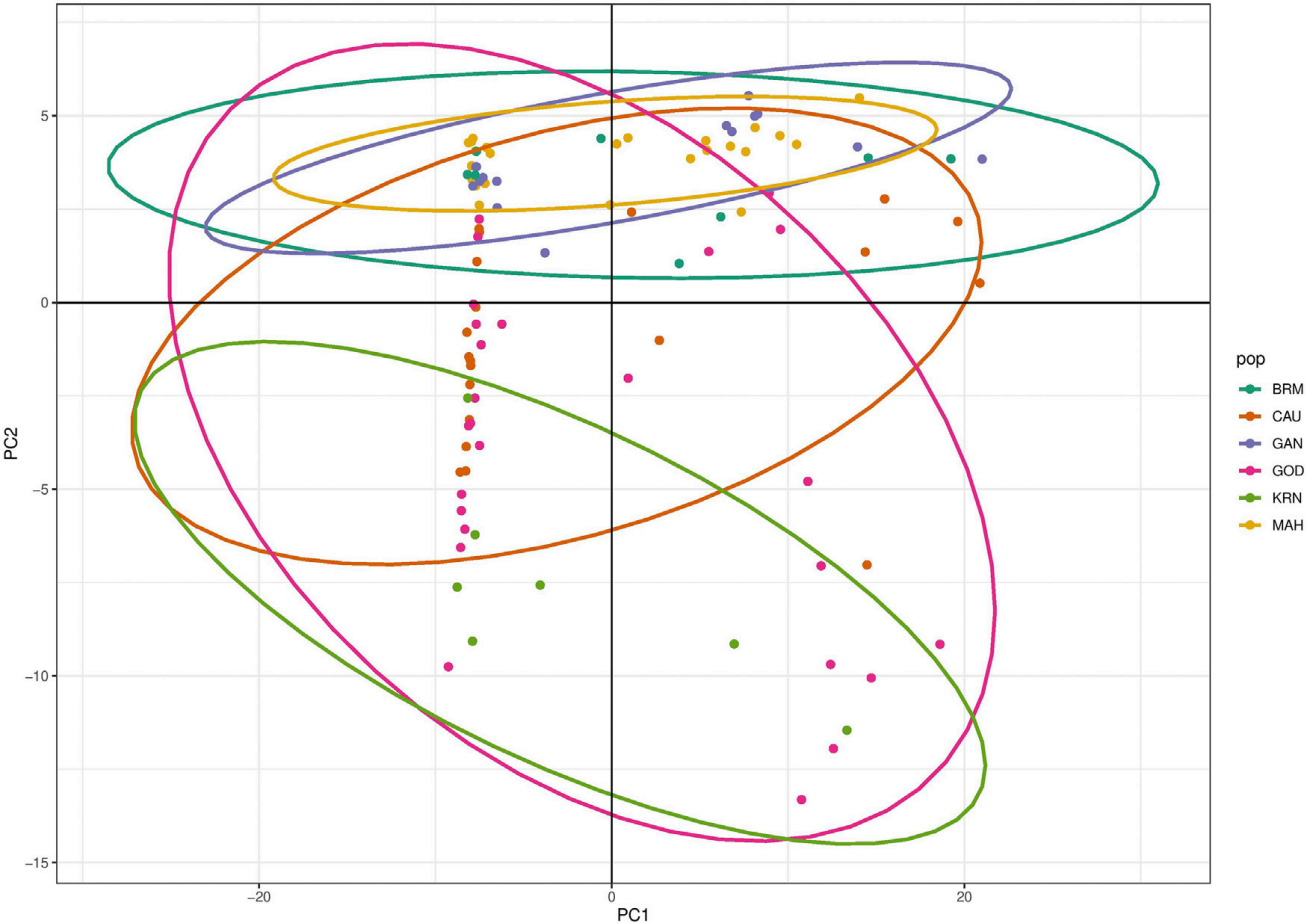
PCA scatter plot showing relationships for six catla populations.

**FIGURE 3:**
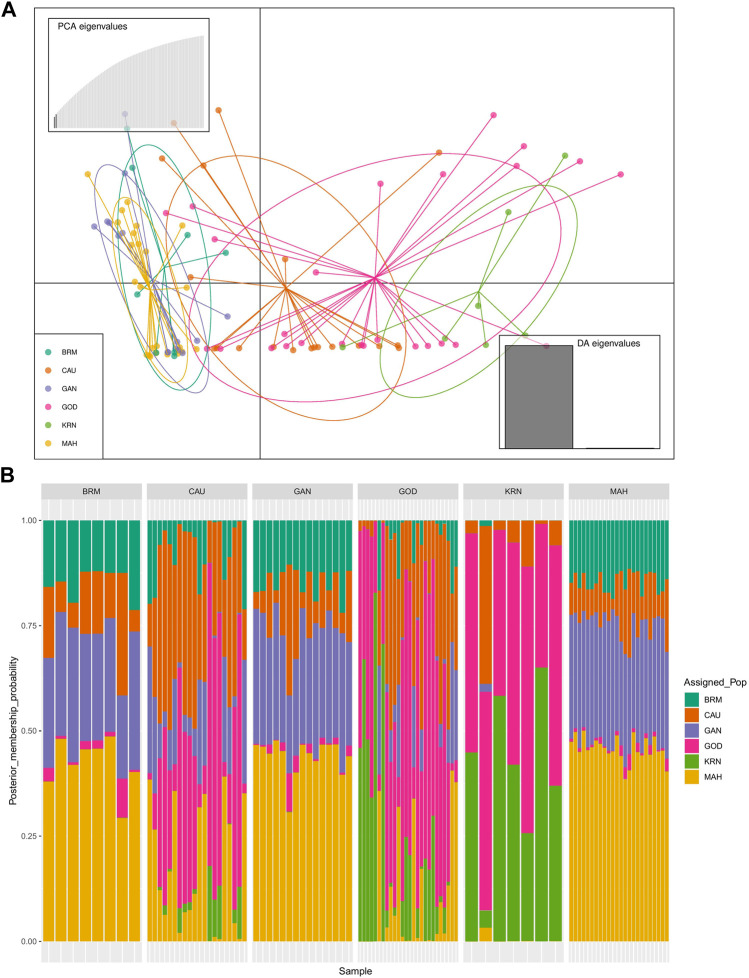
Discriminant analysis of principal components (DAPC) of six catla populations: **(A)** the scatter plot for all data with two linear discriminants explained 89.68% and 10.32% of the variation, respectively. **(B)** The membership probabilities of each individual in the two assigned clusters estimated by DAPC. The vertical bars correspond to the individuals of each population.

The results of PCA scatter plots are shown in Figure 2. Most individuals from GOD and KRN were grouped together with abscissa values less than 0. Most individuals of BRM, MAH, and GAN together with few individuals from CAU were clustered on abscissa values greater than 0, while most individuals of CAU and GOD showed closer affinity toward the KRN cluster. Thereafter, DAPC further revealed the population structure with two main clusters (Figure 3A).

**FIGURE 4:**
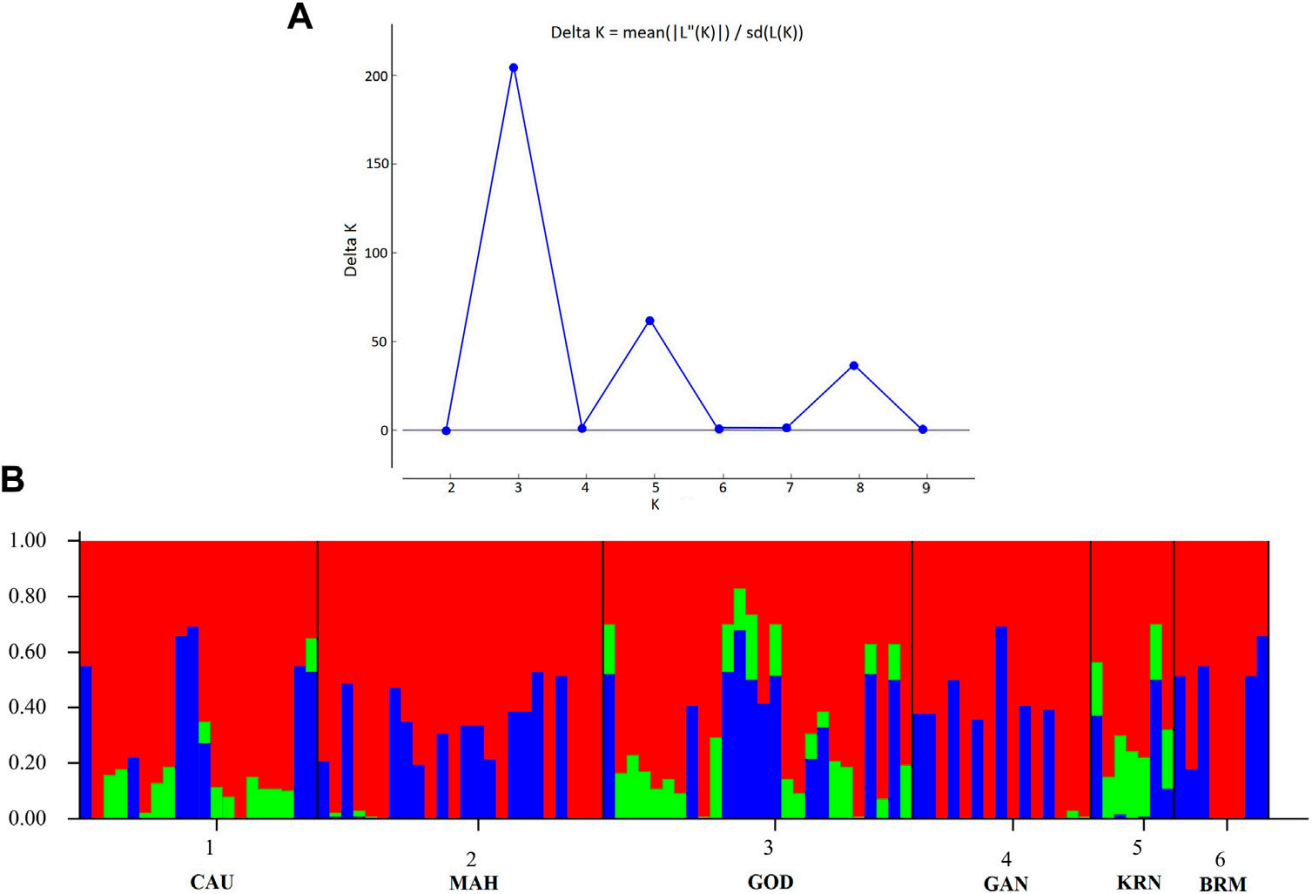
Clustering analysis using STRUCTURE. **(A)** The optimal cluster value (K) was k = 3 with ΔK. **(B)** The Structure analysis with admixture results for K = 3 with two clusters.

In addition, Figure 3B shows the membership probabilities of each individual in various clusters. This bar graph provides a more organized picture of our data by comparing the population membership probability assignments with the populations to which they were first assigned. The individuals of BRM, MAH, and GAN populations were assigned to the same cluster, and most individuals of KRN populations were allocated to another cluster. CAU and GOD, on the other hand, had substantial admixture from all clusters. Furthermore, the Bayesian clustering algorithm STRUCTURE revealed the shared population ancestry of 100 individuals partitioned into two clusters at the optimal cluster value K = 3 (Figures 4A,B).

**FIGURE 5:**
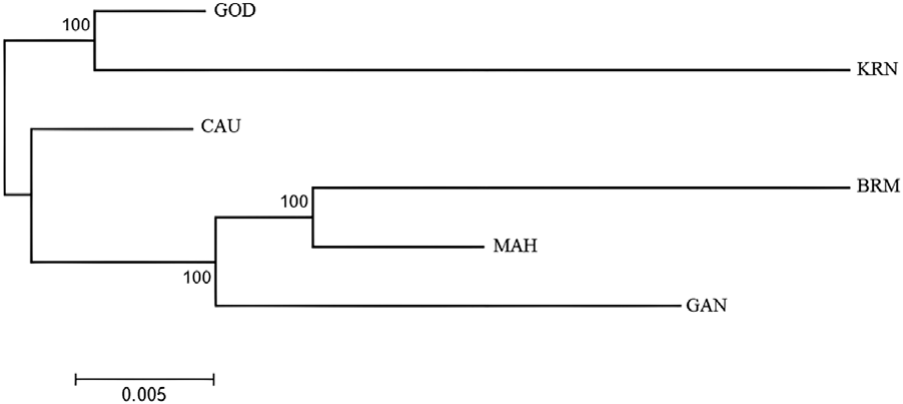
The UPGMA tree of six populations constructed with the ape package.

The UPGMA tree of the six populations (Figure 5) showed two genetically separate clusters, with almost all KRN and GOD individuals clustering together (cluster 1) and the remaining individuals aggregating into a second cluster (cluster 2). Individuals from MAH, BRM, and GAN made up most of the individuals in cluster 2.

### 3.3 Genetic differentiation and gene flow

**FIGURE 6:**
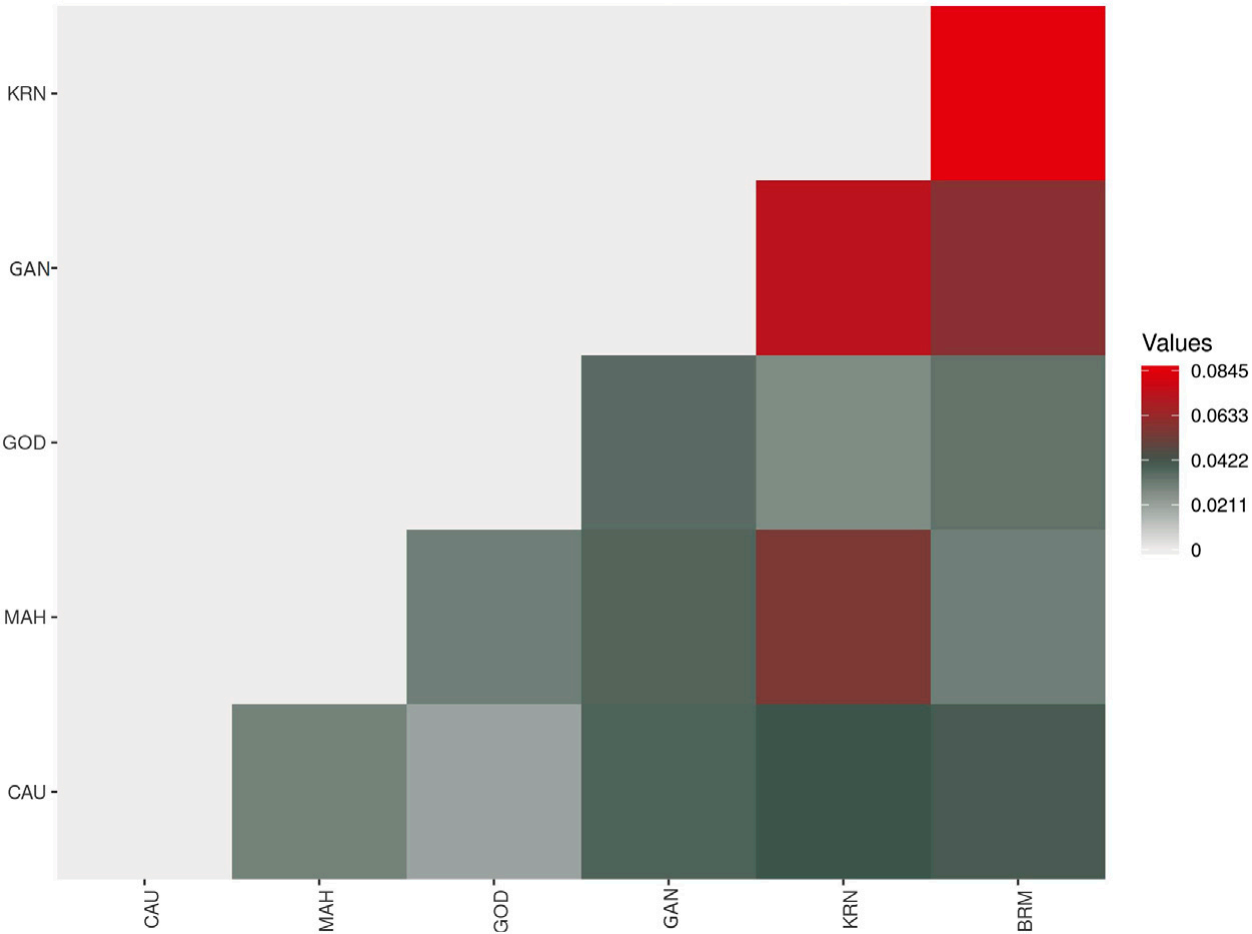
The pairwise F_st_ heatmap showing genetic diversity among six catla populations.

The pairwise F_st_ values ranged from 0.0208 to 0.0845 for these six catla populations (Figure 6; Table 4). The maximum genetic difference (F_st_ = 0.0845) was found between the BRM and KRN populations. In contrast, the CAU and GOD populations had the least genetic difference (F_st_ = 0.0208). Furthermore, to study the genetic variations across the populations, AMOVA (Table 5) was performed. Within populations, variation was found to be higher (95.32%) in proportion. On the other hand, the variation among populations accounted for just 4.68% of the overall molecular variation in the studied populations.

**TABLE 4:** Pairwise F_st_ values among six populations of *Labeo catla*.

Population	CAU	MAH	GOD	GAN	KRN	BRM
CAU	0.000	0.030028	0.020856	0.038303	0.041876	0.04038
MAH		0.000	0.030912	0.037165	0.054916^a^	0.030667
GOD			0.000	0.035488	0.027421	0.03401
GAN				0.000	0.0731^a^	0.058576^a^
KRN					0.000	0.084462^a^

^a^ Significant (F_st_ > 0.05).

**TABLE 5:** AMOVA in six different *Labeo catla* populations.

Source of variation	Degree of freedom	Sum of squares	Variance components	Percentage of variation
Among populations	5	3466.992	13.23174 Va	4.68
Within populations	194	52273.973	269.45347 Vb	95.32
Total	199	55740.965	282.68521	100

Fixation index F_st_: 0.04681.

### 3.4 Linkage disequilibrium

**FIGURE 7:**
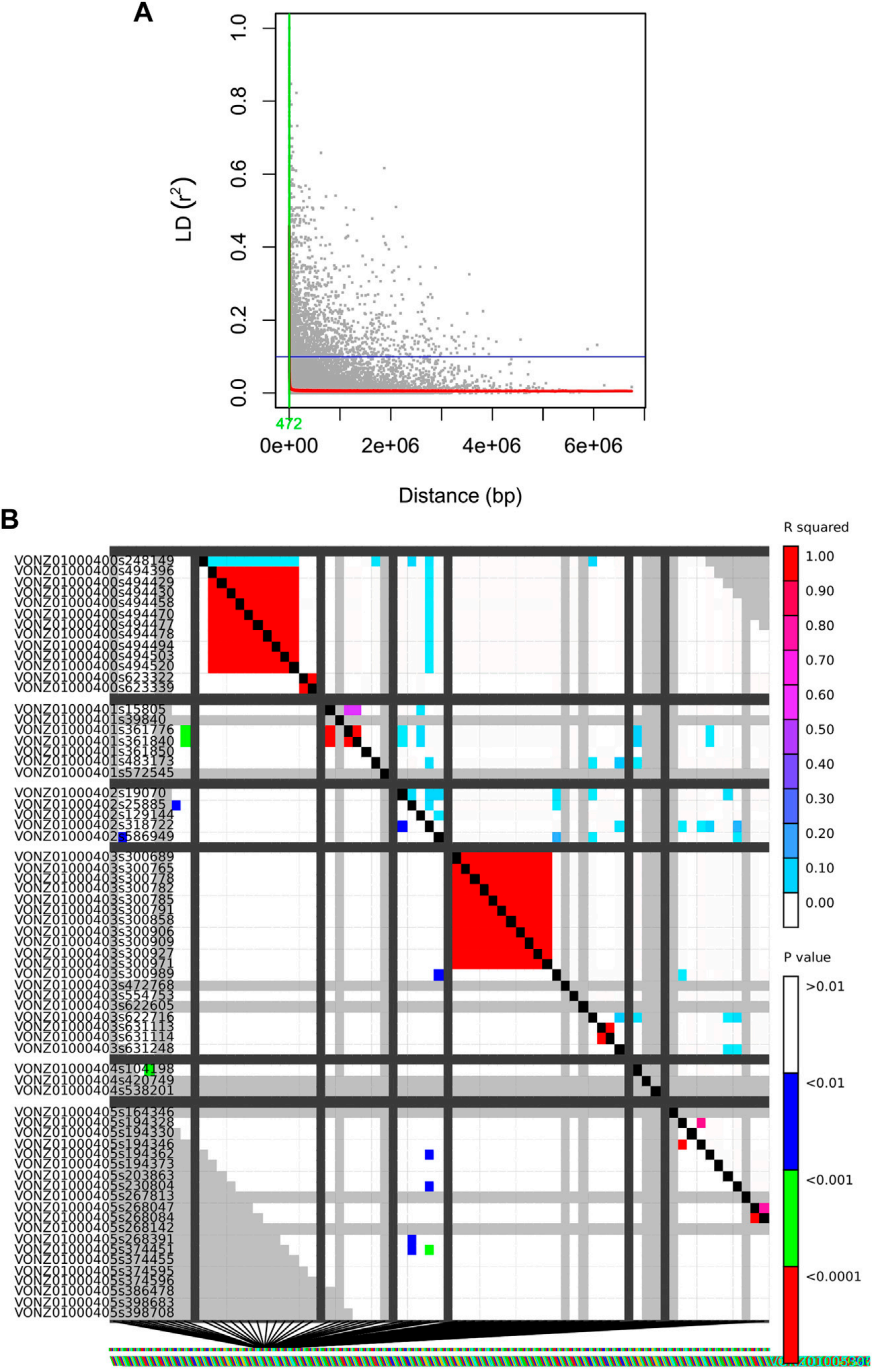
**(A)** Genome-wide LD decay plots. The x-axis shows the distance between SNPs in base pair, while the y-axis indicates the LD values in r^2^. Half LD and LD decay distance are shown by the horizontal lines and vertical lines, respectively. **(B)** The x and y axes are used to illustrate the pairwise LD scores of polymorphism sites. The values for R-squared are shown above the diagonal, while the associated values for P are shown below the diagonal. The results of a comparison between two sets of marker sites are displayed by each cell, with colour coding based on the occurrence of significant LD. In both diagonals, the substantial threshold levels are represented by a coloured barcode.

The whole-genome average maximum r^2^ value was found to be 0.2, which reduced to 0.1 at a distance of 472 bp. Hence, this distance is assumed to represent the whole-genome LD decay distance and LD decayed above this distance (Figure 7). Thus, no significant pairwise linkage disequilibrium was observed among the loci.

## 4 Discussion

### 4.1 SNP identification, genetic diversity, and population structure

Genotyping-by-sequencing resulted in 10,485 putative SNP markers from the 278 million filtered reads. Nucleotide diversity is an important indicator for assessing the DNA sequence diversity of a species or population (Goodall-Copestake et al., 2012). The nucleotide diversity (**π)** that was identified in this research falls within the range that has been reported in earlier studies involving freshwater fishes (Habib et al., 2012; Nyinondi et al., 2020; Zhou et al., 2021). All six catla populations showed relatively low nucleotide diversity, with the GAN population having the lowest value. Fast lineage shortening within founder populations leads to low nucleotide diversity, which is a feature of a narrow genetic structure (Grant and Bowen, 1998). Loss of allelic heterozygosity in our sampling sites may be due to habitat loss and fragmentation that led to inbreeding and selection (Malini and Rao, 2004; Jadhav et al., 2011; Kumar et al., 2020; Das et al., 2021). Another possible explanation for the absence of considerable allelic heterogeneity in the catla population may be the “stepping stone model of migration” (Felsenstein, 1997), which links effective gene flow to gene exchange among neighboring populations (Kumar et al., 2014; Das et al., 2021).

In addition to nucleotide diversity, heterozygosity is also a key determinant of population diversity in a given species. In this study, low levels of heterozygosity were observed across all populations. The observed heterozygosity was lower than the expected heterozygosity, which is similar to the findings of Faroque et al. (2021) and Alam and Islam. (2005) in *L. catla* and Alam et al. (2009) in *L. rohita* using microsatellite DNA markers. However, our result showed a small difference between the mean He and Ho. Observations made by Hamilton et al. (2019) were at most similar for wild populations of catla using SNP markers. This indicates that, over time, the rivers containing wild populations of *L. catla* have experienced a decrease in genetic diversity, but the specific reasons for this are not clear. The loss in river population is thought to be caused by unregulated fishing methods, including the overexploitation of seed, inadequate and inconsistent river flow as a result of the construction of dams, and water contamination by human activities (Alam et al., 2009). Heterozygosity losses may also be the consequence of inbreeding, selection, and decline in breeding populations due to intermixing of diversified genetic stocks or the Wahlund effect (Hartl and Clark, 1997).

For a long time, rivers have been used for industrial development, electricity production, fishing, and agriculture near highly urbanized areas. The major effects of urbanization on river systems include habitat loss and fragmentation, altered water quality (due to an increase in the loading of pollutants, fertilizers, and sediment), and biological invasion (Simpson et al., 2016; Hammerschlag et al., 2017). Similar incidents were observed at our sampling locations. Rapid urbanization around the industrial areas of Sambalpur and Cuttack near the Mahanadi River has been polluting the river water with sewerage, industrial effluents, and biomedical waste (Swain et al., 2017). Industrial and agricultural runoffs (Nath et al., 2018), as well as effluents from the Rourkela Steel Plant and other chemical industries in the river bed, moderately affect Brahmani River water quality near Barkote (Swain et al., 2017). Rajahmundry was the only sampling site where the *Water Quality Index (WQI)* of the Godavari River was found to be fair and less environmental impact was noted (Sravya et al., 2017). According to Susheela et al. (2014), the Cauvery River at Mysore has an alarming level of metal pollution and altered water quality due to nearby agricultural and industrial activities and a damming effect due to the presence of the Krishna Raja Sagara Dam. Additionally, the Ganga River at Patna recorded a colossal level of urbanization (Imam, 2020) along with air and water pollution as a result of rampant sand mining and brick kilns. This may be the reason for the lower diversity of the GAN population compared with other sampled wild populations. At the same time, compromised water quality due to fly ash arising from the Vijayawada Thermal Power Station has been reported (Rao and Ravindhranath, 2013). Also, the fish fauna of the Krishna River near the Prakasam Barrage is in danger due to a number of issues, such as intensive fishing, competition and predation from invasive species, and habitat degradation from pollution (Jadhav et al., 2011). Dam-induced delta erosion affects estuarine fisheries and reduces the biodiversity of estuarine flora and fauna, which leads to effects on the effective population size and inbreeding levels (Malini and Rao, 2004). Habitat loss, fragmentation, and hydrological alteration arise from excessive sand mining, encroachment, and siltation on river beds of the Saruali and Rengali dams (Das et al., 2021). An observation on the migration of fish from their natural habitat resulting in inbreeding and selection due to the presence of the Hirakud Dam has been reported (Kumar et al., 2020), which is a major concern. The absence of freshwater in the estuaries and the destruction of mangrove forests have caused a decline in fisheries in the Cauvery, Krishna, Godavari, Mahanadi, and Brahmani estuaries. Salinity variations brought on by water obstruction (Dandekar, 2012) and biological invasion of alien fish due to the Krishna Godavari River linking project at Vijayawada in Nagarjuna Sagar Dam, Rajahmundry in the Dowleswaram Barrage, and dams at Patna (Dandekar, 2012; Singh et al., 2013; Babu and Padmavathi, 2016) are also affecting fish heterozygosity and nucleotide diversity.

The inbreeding coefficients (F_IS_) of six populations of catla indicate increased homozygosity in GOD, as compared to other studied wild populations (Table 3). The occurrence of non-random mating or population division is frequently indicated by high F_IS_ readings (Allendorf et al., 2007). In this investigation, the resulting alleles were found to be polymorphic (Table 3). When the most frequent allele’s frequency was less than or equal to 0.99, then that region was defined as a polymorphic locus (Hartl and Clark, 1997). The observed unexpected maximum private allele in the GAN population at any SNP loci argues against the effective ongoing gene flow. This unexpected outcome may be due to the small sampling size or it may show maximum differentiation within a population due to the random mutation (Hartl and Clark, 1997), which thus requires further investigation.

Effective population size (Ne), usually less than the census population size, influences genetic diversity in a population. Our results showed lower levels of Ne ranging from 0.8 to 1.2, and similar observations were reported by Escalante et al. (2020) and Diaz et al. (2021). Inbreeding and genetic drift are expected to reduce genetic diversity as Ne decreases (Frankham, 2005). In this study, the CAU population had the lowest Ne (0.8) with relatively lower nucleotide diversity (0.178) and observed heterozygosity (0.055) than all other populations, resulting in a moderately higher F_IS_ value (0.57). However, this is not always true in populations in which genetic diversity is altered by external influences. For example, despite decreases in Ne (0.9) in the GOD population, there was no decline in the nucleotide diversity (0.205) and observed heterozygosity (0.059). Despite having a higher F_IS_ value (0.637), genetic diversity seemed to be maintained through immigration and other events, such as the recent artificial interlinking of rivers. A similar observation was obtained in Atlantic salmon (*Salmo salar*) (Consuegra et al., 2005; Fraser et al., 2007). In addition to this, the GOD population at Rajahmundry has been reported to have a fair *Water Quality Index* (*WQI*) and lower pollution levels (Sravya et al., 2017), indicating less influence on genetic diversity. The BRM population had a higher level of Ne (1.1), indicating higher genetic diversity. Estimated genetic indices of the BRM population showed a similar pattern, with the highest Ho (0.06) among populations under study. Similar findings were obtained for the KRN population, which had the highest effective population size (1.2) with a higher Ho (0.059) among populations. The most concerning populations in our studies were those of Mahanadi and Ganga. Among six populations, the GAN population had the lowest Ne (0.9), the lowest H_o_ (0.053), and the lowest nucleotide diversity (0.168) with a moderately higher inbreeding (0.428) value. Similar observations were seen in the MAH population, which had a relatively lower Ne (0.9) and a lower nucleotide diversity (0.174) and H_o_ (0.054). The observed reduction in heterozygosity could be attributed to the enormous pollution due to industrial activities, sand mining, and the release of agricultural runoffs in the Ganga River (Imam, 2020). In addition, activities related to coal mines and water pollution by sewerage and biomedical waste from two highly urbanized industrial cities such as Sambalpur and Cuttack have adversely affected the genetic diversity in the MAH population. Furthermore, habitat fragmentation due to the presence of the Hirakud Dam has also been reported (Swain et al., 2017). Thus, GAN and MAH populations require attention and efficient management for the conservation of wild populations of catla.

In this study, the PCA indicated no substantial grouping by populations or sampling regions as these samples of catla populations, to some extent, were leading to the probability of gene pool mixing as a result of anthropogenic activities. Specific reasons in relation to the anthropogenic activities can be attributed to rigorous aquaculture activities, such as seed production in hatcheries, in which unregulated hybrid fish production might pose a threat in terms of genetic pool contamination. Another major concern in the Indian context is the significant inbreeding rate in hatcheries. Hatchery-produced inbred individuals escaping to the natural environment may also cause the gene pool to be adversely affected (Alam et al., 2009; Mandal et al., 2014). Nevertheless, it seems that there are two major clusters: the first cluster contains GOD and KRN, while the second cluster has the other remaining populations. The results of the PCA and DAPC can be seen to be very similar, with an organized picture created using the membership probabilities of each individual in the DAPC, in which the individuals from the BRM, MAH, and GAN populations are all in the same cluster, while the individuals from the KRN population fall into a separate cluster and individuals from the CAU and GOD populations are admixed. The overlap in clusters and the lack of significant distances between groups shown by the multivariate DAPC may be explained by high gene flow and the mixing events that have taken place in individuals within the populations under study. Traditionally, for structure analysis, the most probable K is chosen by applying the highest value of L(K), but in the majority of cases, L(K) tends to marginally rise even after the genuine K has been attained. As a result, we computed ΔK according to Evanno et al. (2005) as well. At the most likely value of K, a clearer peak can be presented using ΔK (Figure 4A). Ancestral proportions at K = 3 indicated that the 100 individuals were partitioned into two clusters (Figure 4B). The STRUCTURE analysis result is similar to the DAPC result, which shows that six populations were divided into two main clusters. No significant population structure was observed in catla, as reported by Hamilton et al. (2019) and Das Mahapatra et al. (2018). Furthermore, ancestry analysis revealed the occurrence of mixing among the aforementioned groups. This could be due to the intermixing of individuals between rivers through human intervention. Another possible explanation for this phenomenon might be that the *L. catla* species taken from distinct river basins in the current study had a similar ancestral gene pool, for which at least historical connections existed across different rivers (Daniels, 2001). The distribution of river systems has the greatest influence on the population structure of freshwater species. The result of the UPGMA tree of the six populations of catla also showed two genetic clusters among the six populations, which was in line with the results of the PCA and DAPC. Similar results were shown in *L. catla* (Rahman et al., 2009; Ali et al., 2015) and *L. rohita* (Islam and Alam, 2004) using RAPD analysis, and also in *L. rohita* (Alam et al., 2009) using microsatellite DNA markers among the Halda, Padma, and Jamuna rivers, respectively.

In brief, the six populations of *L. catla* were divided into two clusters based on the results of PCA, DAPC, STRUCTURE, and the UPGMA tree, with a moderate degree of genetic divergence. According to the outcomes of genetic admixture and phylogenetic analysis, a significant number of individuals from the BRM, MAH, and GAN populations of catla were clustered together and it was not possible to entirely differentiate the two clusters due to the presence of genetic overlap. In particular, the majority of individuals originating from GOD populations displayed a closer kinship to the KRN populations.

### 4.2 Population genetic differentiation and gene flow

The pairwise F_st_ values ranged from 0.0208 to 0.0845 for these six catla populations, showing low-to-moderate levels of genetic differentiation. A similar observation was made by Das et al. (2012) using mtDNA markers; low SNP marker diversity and lack of genetic differentiation were reported by Hamilton et al. (2019). In general, F_st_ values ranging from 0.05 to 0.15 suggest a moderate level of genetic differentiation between populations (Wright, 1978). The maximum genetic difference (F_st_ = 0.0845) was found between the BRM and KRN populations. In contrast, the CAU and GOD populations had the least genetic difference (F_st_ = 0.0208). The F_st_ value revealed that KRN samples were significantly different from the BRM, GAN, and MAH samples, whereas BRM samples were observed to be substantially different from GAN. Smaller genetic distance and high gene flow might be the possible reasons for reduced genetic differentiation between the CAU and GOD populations. Our sample of SNPs, which shows low-to-moderate molecular marker differentiation across rivers, revealed minimal genetic differentiation because of adaptive selection or drift and suggested that there had been continuous gene flow amongst the analyzed river basins (Hamilton et al., 2019). As with many other Indo-Malayan fish, *L. catla* is thought to have invaded India during the Eocene epoch through the Indo-Brahma River, which flows from Assam to the Arabian Sea in a westerly direction, confirming a common ancestry of catla species in the rivers of India (Daniels, 2001). Additionally, in the lack of connectivity among the rivers, low-to-moderate genetic differentiation is most likely the outcome of gene flow brought about by species introduction into waterways, unethical aquaculture practices, environmental catastrophes, and others. Furthermore, AMOVA results revealed that within-population variation was higher (95.32%) in proportion than that in among-population variation (4.68%), which coincides with the results obtained by Hamilton et al. (2019), Sahoo et al. (2018), and Das et al. (2012), and is possibly because of the increased gene flow among the populations (Slatkin and Barton, 1989; Allendorf et al., 2012). This kind of gene flow may lower genetic differentiation and account for the substantial within-population variability (Ferguson et al., 1995), as seen in *L. catla* samples from six river basins. The fixation index (Fst = 0.04681; *p* = 0.00000 ± 0.00000) revealed minimal genetic variation among populations (Weir and Cockerham, 1984; Excoffier et al., 1992).

### 4.3 Linkage disequilibrium

In this study, no significant pairwise linkage disequilibrium was observed among the loci, which is similar to the findings obtained by Das Mahaptra et al. (2018). Our results indicated LD to be entirely decayed at r^2^ < ∼ 0.2 as it approaches equilibrium. This, along with LD decay at r^2^ = half decay distance, is a widely used criterion in the literature (Hudson, 2004; VanLiere and Rosenberg, 2008; Vos et al., 2017). An admixed population structure can sometimes lead to reduced levels of linkage disequilibrium (LD) between genetic variants. An admixed population is a population that has arisen from the interbreeding of two or more ancestral populations. As per our result, LD between these variants may be reduced or even eliminated in the admixed population. Furthermore, it is important to note that while admixture can lead to reduced levels of LD between certain genetic variants, the effects of admixture on LD can be complex and context-dependent.

## 5 Conclusion

In the present investigation, 10,485 genome-wide SNPs were identified using the GBS method for genome-scale analysis in six riverine populations of *L. catla*. Low genetic diversity was observed with low-to-moderate levels of pairwise genetic differentiation in the six catla populations. Most of the genetic variations were found within populations. Both Bayesian and multivariate techniques divided *L. catla* into two clusters. The results of this study will help in the conservation and management of wild populations of catla.

## Data Availability

The datasets presented in this study can be found in online repositories. The names of the repository/repositories and accession number(s) can be found at: https://www.ncbi.nlm.nih.gov/, PRJNA886474.
