# Supplementary material for: Genetic diversity and genome-scale population structure of wild Indian major carp, Labeo catla (Hamilton, 1822), revealed by genotyping-by-sequencing
Source: Front Genet. 2023 May 9;14:1166385. doi: 10.3389/fgene.2023.1166385 (PMC10204928; doi:10.3389/fgene.2023.1166385)
Supplement: Supplementary file 1 [file DataSheet1.ZIP › Supplementary_files/Table S2.docx]

| **Populations** | **Sample size** | **Ne** | **IC 95%** | |
| --- | --- | --- | --- | --- |
| CAU | 20 | 0.8 | 0.8 | 0.8 |
| KRN | 7 | 1.2 | 1.2 | 1.3 |
| GOD | 26 | 0.9 | 0.9 | 0.9 |
| MAH | 24 | 0.9 | 0.9 | 0.9 |
| BRM | 8 | 1.1 | 1.1 | 1.1 |
| GAN | 15 | 0.9 | 0.9 | 0.9 |

Table S2. Estimates of *Labeo calta* effective population size (Ne)

*95% coefficient of interval for effective population size (IC 95%)
